# Adolescent pregnancy in Sao Tome and Principe: a cross-sectional hospital-based study

**DOI:** 10.1186/s12884-022-04632-z

**Published:** 2022-04-15

**Authors:** Alexandra Vasconcelos, Nelson Bandeira, Swasilanne Sousa, Filomena Pereira, Maria do Ceu Machado

**Affiliations:** 1 Unidade de Clínica Tropical - Global Health and Tropical Medicine (GHTM), Instituto de Higiene e Medicina Tropical (IHMT), Universidade NOVA de Lisboa, Lisbon, Portugal; 2 Hospital Dr. Ayres de Menezes, São Tomé, São Tomé and Príncipe; 3 Faculdade de Medicina de Lisboa, Universidade de Lisboa, Lisbon, Portugal

**Keywords:** Adolescent pregnancy, Sao Tome and Principe, Family planning, Newborns’ danger signs

## Abstract

**Background:**

Pregnancy starts early in Sao Tome and Principe (STP) and rates of adolescent pregnancy increased 16% in recent years reaching a 27.3% prevalence. This study aimed to understand the pregnant adolescents’ characteristics and factors associated to early childbearing in STP.

**Methods:**

A cross-sectional hospital-based study was undertaken in Hospital Dr. Ayres de Menezes between 2016 and 2018 with a randomly selected total sample size of 518 mothers. Mothers’ clinical records and interviews were used to collect relevant data. The results among adolescent girls 19 years of age and younger (*n*=104) were compared to adult mothers (*n*=414). A subgroup analysis of adolescent pregnant girls was also conducted. Statistically significance was considered at a *p*-value ≤0.05. Data were analysed using SPSS software.

**Results:**

The study revealed that 20.1% were adolescent mothers. Pregnancy at a very early age (≤15) was experienced by 7.7%. The characteristics founded to be positively associated with adolescent pregnancy were: 1) being single (OR 0.39, 95% CI=0.2–0.6, *p*≤0.001); 2) having a relationship with the baby´s father for a period of less than one year (OR 0.16, 95% CI=0.09-0.3, *p*≤0.001); 3) lack of the baby´s father support (OR 0.41, 95% CI=0.2–0.7, *p*=0.002); 4) not using a contraceptive method (OR 0.33, 95% CI=0.2–0.5, *p*≤0.001), and 5) inappropriate knowledge concerning the identification of the newborn’s danger signs (OR 15.7, 95% CI= 9–26, *p*≤0.001). Comparing pregnancy at very early age (≤15) to late (>18 and ≤19) adolescents, main differences were that previous contraceptives were not used at all in girls ≤15 years compared to 9.8% of late childbearing subgroup.

**Conclusions:**

Unfavourable factors linked to adolescent pregnancies were absence of a contraceptive method, getting pregnant in the early first months of one relationship and to be single. Gap age difference between adolescents’ partners, polygamous sexual relationships, previous abortion and having already other living children were also identified. Adolescents also had inappropriate knowledge of the identification of the newborns’ danger signs. Before being sexually active, adolescents critically need sexual and reproductive health information provided by a healthy community and through school programmes on sexual education. Schools should promote girl’s empowerment and awareness and, at the same time, reinforce boy’s role in fatherhood and shared responsibilities. The government should work on the prevention of early sexual initiation, as well as on improving family planning programmes to protect them from pregnancy with special focus for the very early adolescent girls. None of these goals can be achieved if the government doesn’t, simultaneously, improve educational and economic opportunities for girls.

## Background

Adolescence is a critical phase for a successful transition to adulthood, that is threatened by becoming pregnant under 19 years old as it can greatly alter young women´s life prospects and those of their children [1, 2]. Adolescent pregnancies are a global problem as babies born to adolescent girls account for 11% of all births, with 95% occurring in developing countries [3]. Worldwide, adolescent pregnancies are frequently unplanned, unwanted, and out of wedlock that blocks girls’ education and their social relationships due to stigmatisation and loss of self-esteem, compromising the adolescents’ economic independence [1, 3–7]. Consequently, early motherhood impacts not only girls’ mental and physical health, but it also promotes an intergenerational perpetuation of a poverty trap and cycle [1, 2]. Thus, helping adolescent girls to avoid facing childbearing can have far-reaching benefits for them, their children, and societies [1, 3–7].

Recognizing the adolescents’ vulnerabilities is the first step in approaching this problem. In Africa, according to Kassa et al. 2018 meta-analysis [8], girls were more likely to start childbearing if they were from rural residence, married, not educated, whose parents had no education, and from families that lack parent to child communication on sexual reproductive health issues. Similar findings were also published by Ahinkorah et al. 2021 multi-country analysis [9], except for the finding that girls who lived in rural areas had lower odds of first adolescent pregnancy [9].

Delaying childbearing requires focusing on its major underlying factors: timing of first sex and ineffective contraceptive use, usually associated to girls’ inadequate sexual knowledge; low risk perceptions; ambivalence towards sex; lack of power to negotiate safer sex options and to deal with older partner´s persuasive techniques towards sex [10–13]. Moreover, adolescent girls face a higher risk for unsafe abortion and to be a second time young mothers [10, 14, 15]. Pregnancies complications are more frequent, and babies of adolescent mothers are at a higher risk of being born premature, dying in the neonatal period [16] and facing malnutrition, low mental and physical development, inappropriate social connection with parents and poor education [11, 17, 18]. In general, these risks are lower for adolescents in their late teens but those who give birth before age 15 are at much higher risk [10, 19]. Additionally, the younger the girl the less likely is for her to attend antenatal healthcare as coverage among younger adolescents are lower when compared to older adolescents (18-19 years old) [20] (https://data.unicef.org/topic/child-health/adolescent-health/).

In Africa, this problem is even more hazardous as 33% of girls are giving birth before the age of 18 and 3.5% even before the age of 15 years old [3, 21–23].

Furthermore, it is foreseen that the percentage of adolescent pregnancies will increase globally by 2030, particularly in the Sub-Saharan African countries [22]. These can be due to girls’ low economic status, poor living arrangements, early school drop-out, inappropriate knowledge of sexual and reproductive health issues, absence of family planning, lack of sexuality education in schools and low employment attainment [8].

This calls for efforts to address the sexual and reproductive health problems of adolescent girls, included for the first time in the Sustainable Development Goals (SDGs) Countdown to 2030 [24, 25]. First step should start in the empowerment of girls attending school, preventing their drop-out and giving them access to sexual education with the necessary skills to prevent pregnancy [8].

The Democratic Republic of Sao Tome and Principe (STP), two islands in the Gulf of Guinea, has one of the highest rates of teenage pregnancies in sub-Saharan Africa, estimated at about 27% [26, 27]. Pregnancy starts early in STP and adolescent pregnancy rate in the past years increased by 16% (22.8% in 2009 to 27.3% in 2014). With a population of about 200.000, STP has a high adolescent fertility rate of 94.8 births per 1,000 women aged 15 to 19 years compared for example to 68 in South Africa or 61 in Sudan [23, 27–30]. Although, adolescent pregnancy is recognized as a major public health issue in the country and the government seeks to be able to reduce it to a rate of 15% [28, 31–36] (https://www.un.org/en/development/desa/population/publications/pdf/popfacts/PopFacts_2019-1.pdf), it has not been yet properly studied [21, 31–33, 37].

This study therefore sought to investigate the differences between adolescents and older women in relation to selected sociodemographic and family characteristics, obstetric history, and knowledge of the identification of the newborns’ danger signs. We also aimed to explore the eventual differences of “very early childbearing” defined as birth before age ≤15, early childbearing (birth between age >16 and ≤17) and late childbearing (birth age >18 and ≤19) in comparison to the older women group trying to figure out main teen’s vulnerabilities.

We have previously studied these adolescent girls regarding their adverse outcomes and founded that foetal distress and the need for performance of neonatal resuscitation manoeuvres were the main adverse perinatal outcomes imputable to these adolescent births.

We believe that understanding the characteristics of pregnant adolescents and risk factors that promote early childbearing will support STP policy makers in order to design pragmatic interventional programs to reduce the countries overwhelming high rates of adolescent pregnancies and achieving key SDG [34].

## Material and methods

### Study design

This is a cross-sectional analytical hospital-based study conducted between July 2016 to November 2018.

### Study context

The study was located at Hospital Dr. Ayres de Menezes (HAM) in Sao Tome city, the capital of STP, a rapidly growing city, with 64% of the population living in the capital [33]. HAM is the only hospital in the island, with a maternity that delivers around 4700 babies annually, around 82,4% of all the deliveries in the country [32]. Comprehensive Emergency Obstetric and Neonatal Care Services are available at HAM. There are 6 health centres with maternities in the rural area.

### Study population and sampling

Mothers were randomly selected by chance and the study was done in different months to avoid seasonal interference and effects by means of confounding variables by guaranteeing a sample with few biases. Two groups were considered, over or under and equal to 19 years of age. Of the total 518 pregnancies, 104 were in women aged ≤19 years (adolescent group) and 414 pregnancies in women more than 19 years of age (older childbearer group). For some variables (Tables 1, 2 and 4) further analyses with three adolescents age subgroups were used for the purpose of finding eventual differences of “very early childbearing” as birth before age ≤15 (*n*= 8), early childbearing for birth between age >16 and ≤17 (*n*=45) and late childbearing for birth age >18 and ≤19 (*n*=51) in comparison to the older women group.

**Table 1:** Socio-demographic characteristics for different adolescent subgroups and total adolescent pregnant girls in comparison to the older women group

Characteristics	Pregnancies ≤19 years old				Pregnancies >20 years old
	very early childbearing (≤15) *n*= 8 (%)	early childbearing (≥16 and ≤17) *n*=45 (%)	late childbearing (≥18 and ≤19) *n*=51 (%)	Total adolescents ***n***= 104 (%)	***n***=414 (%)
**Residence**					
rural	6 (75%)	30 (67%)	27 (52.9%)	63 (60.6%)	213 (51.4%)
urban	1 (12.5%)	15 (33%)	22 (43.2%)	38 (36.5%)	194 (46.9%)
missings	1 (12.5%)	-	2 (3.9%)	3 (2.9%)	7 (1.7%)
OR and 95% CI	0.1 (0.02-1.5)	0.5 (0.29-1.06)	0.8 (0.48-1.59)	0.66 (0.4-1.03)	
*p* value	0.11	0.07	0.66	0.07	
**Mother´s education level**					
elementary only	5 (62.5%)	20 (44%)	19 (37.3%)	44 (42.3%)	160 (38.6%)
secondary	3 (37.5%)	25 (56%)	32 (62.7%)	60 (57.9%)	254 (61.4%)
OR and 95% CI	2.8 (0.66-11.9)	1.4 (0.75-2.63)	0.9 (0.5-1.8)	1.3 (0.9-2)	
*p* value	0.16	0.29	0.99	0.19	
**Employed**					
no	5 (62.5%)	31 (69%)	32 (64%)	68 (65.4%)	262 (63.3%)
yes	-	1 (2%)	7 (14%)	8 (7.7%)	134 (32.4%)
missings	-	-	1 (6.3%)	1 (0.9%)	9 (2.2%)
OR and 95% CI	-	-	-	0.17 (0.8-0.37)	
*p* value	-	-	-	≤0.001*	
**Still studying**					
yes	3 (37.5%)	13 (29%)	11 (22%)	27 (26%)	9 (2.2%)
OR and 95% CI	-	-	-	15.8 (7-35)	
*p* value	-	-	-	≤0.001**	
**Married**					
no	6 (75%)	14 (31%)	13 (25.5%)	33 (31.7%)	59 (14.3%)
yes	2 (25%)	31 (69%)	37 (72.5%)	70 (67.3%)	336 (81.2%)
missings	-	-	1 (2%)	1 (1%)	19 (4.6%)
OR and 95% CI	0.05 (0.01-0.2)	0.34 (0.16-0.7)	0.46 (0.23-0.9)	0.39 (0.2-0.6)	
*p* value	≤0.001	0.003	0.03	≤0.001***	
**Babies’ father support**					
yes	6 (75%)	34 (76%)	44 (86.3%)	84 (80.7%)	373 (90.1%)
no	2 (25%)	11 (24%)	7 (13.7%)	20 (19.2%)	41 (9.9%)
OR and 95% CI	0.3 (0.06-1.7)	0.34 (0.1-0.7)	0.7 (0.29-1.6)	0.41 (0.2-0.7)	
*p* value	0.19	0.005	0.42	0.002****	
**Father´s age**					
<20 years old	2 (25%)	11 (24%)	4 (9.1%)	17 (16.3%)	2 (0.5%)
20-29	5 (62.5%)	27 (60%)	33 (75%)	65 (62.5%)	124 (30%)
30-39	-	3 (7%)	7 (15.9%)	10 (9.6%)	161 (38.9%)
40-49	-	-	-	-	64 (15.5%)
>49	-	-	-	-	25 (6%)
missing	1 (9.2%)	4 (9%)	7 (13.7%)	12 (11.5%)	38 (9.2%)
OR and 95% CI	-	-	-	-	
p value	-	-	-	-	
**Father´s education level**					
elementary only	3 (37.5%)	9 (20%)	16 (31.4%)	28 (26.9%)	129 (31.2%)
secondary	3 (37.5%)	15 (33%)	19 (37.2%)	37 (35.6%)	151 (36.5%)
doesn´t know/missing^ɫ^	2 (25%)	21 (47%)	16 (31.4%)	39 (37.5%)	134 (32.4%)
OR and 95% CI	-	-	-	-	
p value	-	-	-	-	

*Abbreviations*: *OR* Odds Ratio, *CI* Confidence interval

* The adolescent group reported to have a job in 7.7% compared to a higher proportion of 32.4% for the older counterpart with a significant statistical difference (OR 0.17, 95% CI=0.8-0.37, *p*≤ 0.001).

** There was a higher proportion of adolescent mothers who were students at the time they got pregnant, with a statistically significant difference (OR 15.8, 95% CI=7-35, *p*≤0.001).

***67.3% of the teenage girls were married and 31.7% were single compared to 81.2% and 14.3% in the older women group, which was statistically significant (OR 0.39, 95% CI=0.2-0.6, *p*≤0.001).

**** The adolescent´s pregnancy was not supported/followed by the baby´s father in 20% of the cases compared to 9.9% of the older women group, the difference being statistically significant (OR=0.41, 95% CI=0.2–0.7, *p*=0.002).

^ɫ^Most adolescents didn´t answer because they were not aware of the fathers’ education level achieved

**Table 2:** Obstetric history, ANC and family planning for the different adolescent subgroups in comparison to the older women group

Characteristics	Pregnancies ≤19 years old				Pregnancies >20 years old
	very early childbearing (≤15) *n*= 8 (%)	early childbearing (≥16 and ≤17) *n*=45 (%)	late childbearing (≥18 and ≤19) *n*=51 (%)	Total adolescents ***n***= 104 (%)	***n***=414 (%)
**Previous contraceptive use**					
yes	0 (0%)	2 (4%)	5 (9.8%)	7 (6.7%)	119 (28.7%)
no	7 (87.5%)	36 (80%)	41 (80.3%)	84 (80.7%)	217 (52.4%)
missings^#^	1 (12.5%)	7 (16%)	5 (9.8%)	13 (12.6%)	78 (18.8%)
OR and 95% CI	-	0.14 (0.03-0.59)	0.24 (0.08-0.6)	0.33 (0.2-0.5)	
*p* value	-	0.007	0.007	≤0.001*	
**Pregnancy was planned**					
yes	0 (0%)	5 (11%)	14 (27.4%)	19 (18.3%)	102 (24.6%)
no	5 (62.5%)	30 (67%)	25 (49%)	60 (57.7%)	231 (55.8%)
missings^#^	3 (37.5%)	10 (22%)	12 (23.5%)	25 (24%)	81 (19.6%)
OR and 95% CI	-	0.39 (0.1-1.0)	1.2 (0.6-2.5)	-	
*p* value	-	0.06	0.49	-	
**Gravidity**					
1	7 (87.5%)	39 (87%)	27 (52.9%)	73 (70.2%)	54 (13%)
2	1 (12.5%)	5 (11%)	19 (37.3%)	25 (24%)	78 (18.8%)
3 or more	0 (0%)	1 (2%)	5 (9.8%)	6 (5.8%)	282 (68.1%)
OR and 95% CI				17.7 (9-26)	
p value				≤0.001**	
**Abortion/ miscarriage**					
0	7 (87.5%)	41 (91%)	41 (82%)	89 (85.5%)	274 (66.2%)
1	1 (12.5%)	4 (9%)	9 (18%)	14 (13.5%)	105 (25.4%)
2	0 (0%)	0 (0%)	0 (0%)	0	22 (5.3%)
>3	0 (0%)	0 (0%)	0 (0%)	0	13 (3.1%)
OR and 95% CI				-	
p value				-	
**Living children**					
0	8 (100%)	43 (96%)	35 (68.6%)	86 (82.7%)	74 (17.9%)
1	0 (0%)	2 (4%)	13 (25.5%)	15 (14.4%)	89 (21.5%)
2	0 (0%)	0 (0%)	3 (5.9%)	3 (2.9%)	97 (23.4%)
>3	0 (0%)	0 (0%)	0 (0%)	0	154 (37.2%)
OR and 95% CI				-	
*p* value				-	
**1st attendance ANC**					
<12th	3 (37.5%)	18 (40%)	34 (66.6%)	55 (52.9%)	218 (52.7%)
>12th	3 (37.5%)	22 (49%)	12	37 (35.6%)	136 (32.8%)
missings	2 (25%)	5 (11%)	5 (9.8%)	12 (11.5%)	60 (14.5%)
OR and 95% CI	0.8 (0.17-4.3)	0.7 (0.3-1.4)	2.47 (1.2-4.9)	-	
*p* value	0.87	0.39	0.01	-	
**ANC**					
at least once				104 (100%)	413 (99.7%)
<4 visits	2 (25%)	8 (18%)	5 (9.8%)	15 (14.4%)	55 (13.3%)
4-7 visits	4 (50%)	21 (47%)	20 (39.2%)	45 (43.3%)	196 (47.3%)
>8 visits	2 (25%)	16 (36%)	26 (51%)	44 (42.3%)	163 (39.4%)
OR and 95% CI				-	
*p* value				-	
**Want to do family planning**					
yes	1 (12.5%)	34 (76%)	16 (31.3%)	51 (49%)	165 (39.9%)
no	0 (0%)	1 (2%)	3 (5.9%)	4 (3.8%)	39 (9.4%)
missings^#^	7 (87.5%)	10 (22%)	32 (62.8%)	49 (47.1%)	194 (46.9%)
OR and 95% CI				0.66 (0.4-1.03)	
*p* value				0.07	

# as don’t know, doesn’t want to reply

*Abbreviations*: *ANC* Antenatal care, *OR* Odds Ratio, *CI* Confidence interval.

* Previous contraceptive use was stated in 6.7% of the teenage group and in 28.7% of the older counterpart, what is a significant statistically difference (OR =0.33, 95% CI = 0.2–0.5, *p* ≤0.001).

** The proportion of primigravidas among teenagers in relation to those aged >20 years was statistically significant (OR 15.7, 95% CI= 9-26, *p*≤0.001).

### Eligibility criteria

All women admitted to the hospital for delivery with a gestational age of 24 weeks or more were eligible to be enrolled in the study. Those who gave birth outside the hospital but were admitted for postnatal were also included in the study.

### Data collection

A face-to-face interview by the main investigator was conducted with the mothers before discharge to gather information regarding socio-demographic characteristics, antenatal care/obstetric history, and their knowledge of identification of newborns’ danger signs (signs and symptoms of disease). The survey was built in the tool QuickTapSurvey© and mothers’ answers were collected and entered to this app. Data regarding obstetric and antenatal care were also checked and collected from medical records and antenatal care pregnancy booklet into the app survey.

### Variables included in the study

*Sociodemographic characteristics included*: (i) residence, (ii) standard of living, (iii) marital status, (iv) living arrangements, (v) mother’s education level, (vi) mother’s employment status, (vii) baby´s father age, (viii) father´s education level, (ix) father´s employment status and (x) father´s living with other woman/houses according to pregnant women´s answer during the interview.

Residence was grouped into urban and rural, urban residence considered for women living at the capital city (Água Grande) and rural in all other districts.

Standard of living was categorized according to household deprived or not of electricity, sanitation, drinking water, cooking fuel and type of housing as Sustainable Development Goal guidelines [38].

Marital status considered the two categories yes (married/union) or no if single/never married. Time of the union/marriage was also assessed.

Mother’s and father’s education level was categorized as either elementary only or secondary level. Taking into consideration that not attending secondary school, usually beginning from the age of 12 years old, is considered to have an inappropriate educational level.

Employment status were collected and classified as no if “not employed” or yes if “employed” (lists of jobs were merged) regardless of the type of job.

*Obstetric and antenatal characteristics included*: (i) obstetric history, (ii) unplanned pregnancy, (iii) previous contraceptive use, (iv) family planning after current pregnancy, (v) antenatal care visits.

Antenatal care (ANC) consisting of less than 4 of expected consultations during pregnancy was considered as inadequate, between 4-7 as adequate and 8 or more visits as complete.

Gravidity categorized as primigravida (1), multigravida (2-4) and grandmultigravida (5 or above).

In each survey, women were asked about previous contraception use and about their future desire to have a contraception method after the pregnancy including the type of contraceptive method they wish to use. We considered as traditional contraceptives the following methods: rhythm, calendar, withdrawal, lactation amenorrhea and “other traditional” methods. As modern contraceptives, we included intrauterine devices and systems, subdermal implants, oral contraceptives, injectable, diaphragms and cervical caps, condoms (male and female), patch, emergency contraception and sterilization.

*Mother´s knowledge of identification of newborns’ danger signs*: was assessed during the interview as “yes” or “no” answer grouped and organized according to the definition presented by the WHO and the Young Infants Clinical Signs study group [39, 40] to the following questions: “Do you think the following are danger signs in the newborn?” (i) convulsions/spasms/rigidity; (ii) difficult/fast breathing; (iii) very small baby (less than 2.5 kg) or losing weight in the first weeks of life; (iv) lethargy/unconsciousness; (v) fever; that is high body temperature that makes the newborn body hot. A score of 3 or more “yes”answers was considered as appropriate knowledge and less than 2 “yes” answers as inappropriate. Locally appropriate and known terms in the appropriate cultural area were used for clinical simplicity purpose and mother´s understanding.

### Data management and statistical analysis

The data were secured in a confidential and private location. Participants were referred to by identification numbers and the informed consent forms were kept separate from the questionnaires. Both could only be linked by a coding sheet available only to the investigators. The software used for sample calculation was Raosoft (http://www.raosoft.com/samplesize.html), but this value was supported by pass software (https://www.ncss.com/software/pass/). There was a mean of 4540 HAM deliveries/year within the study period. The sample was calculated based on a minimum sample of 10% of the population validated by the sample calculation software, which placed the right dimension between 355 (95%) and 579 (99%) confidence. It was possible to collect 518, which gave some comfort at this level. Differences in the demographic and obstetric characteristics between the adolescent and adult mother were assessed using the Pearson’s chi-squared test (χ2). During data analysis, missing values were treated as missing.

The associations between maternal age were categorized as adolescent mothers (1) vs others (0). For each outcome with a statistically significant difference at the first level, further analysis was developed using mothers´ age as a continuous variable with adjustments made simultaneously for father´s support and contraceptive use independently of their statistical significance. For statistical purpose, maternal age was kept as a continuous variable in the models. Mean age of the mothers was 26.59 years (sd 7.1), with a quasi-normal distribution, just underrepresented at the very young mother’s level. Two step logistic regression models were developed to assess the power of age as a predictor. The models were adjusted for marital status, education and antenatal care, the probabilities of event were recorded for each case and the plots obtained with the respective 95% CI for each age point. Regarding confounding variables, multivariate logistic regressions were performed in two stages - first only with the variable under study (mother's age) and afterwards with the inclusion of control variables to understand whether the identified effect occurred via others (education, partner and antenatal care).

Statistical significance was defined as *p*<0.05.

Data were entered in QuickTapSurvey (©2010-2021 Formstack), and the dataset exported to Excel for cleaning and further analysis using the Statistical Package for the Social Sciences for Windows, version 25.0 (IBM Corp. Released 2017. IBM SPSS Statistics for Windows, Version 25.0. Armonk, NY: IBM Corp.).

### Ethics approval and consent to participate

Ethical clearance was obtained from the Democratic Republic of Sao Tome and Principe Ministry of Health and Hospital Dr. Ayres de Menezes. Written informed consent was obtained from all participants (or their parent or legal guardian in the case of teenage under 16) after the purpose of the research was explained orally by the investigator. This was done only after delivery to reduce coercion due to labour pains. Participation in the survey was voluntary, as participants could decline to participate at any time during the study. All methods were performed in accordance with the relevant guidelines and regulations in practice.

## Results

### Socio-demographic and family characteristics

**Fig. 1:**
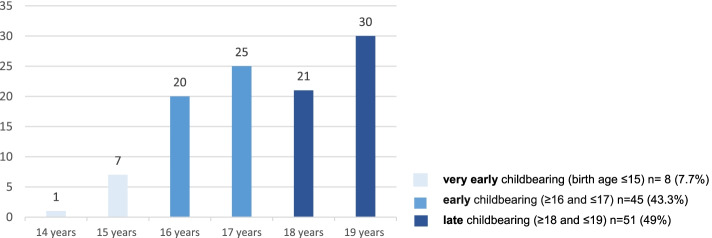
Adolescent ages and childbearing subgroup ages

A total of 518 pregnant women were enrolled. Among the enrolled women, 20.1% (CI – 16,3% to 23%) were adolescents, with 104 teenage pregnancies (≤19 years old) and 414 deliveries in older women. The mean age was 17.42, median 17, with a minimum age 14 years. Regarding childbearing subgroup ages (Fig. 1), 7.7% (8) were very early childbearing adolescents (birth age ≤15), 43.3% (45) were early (≥16 and ≤17) and 49% (51) were late (≥18 and ≤19).

The older women group mean age was 28.9 years, median of 28 with a maximum age of 43 years. Regarding the adult group, the mother´s mean age at first pregnancy was 20.1 (±3.76 SD) years (minimum 13, maximum 36).

Socio-demographic characteristics for the different adolescent subgroups in comparison to the older women group are described in Table 1.

### Marriage, living arrangements and baby´s father support

The duration of the relationship/union or marriage was shorter for the younger ages: 59.5% (62/104) of those were in the union less than one year compared to 19.7% (81/414) of the older counterpart, this was statistically significant (OR 0.16, 95% CI 0.09-0.3, *p* ≤ 0.001). In terms of existence and acknowledgment of a “polygamous” marital informality, 11.6% (12/104) of the adolescent girls reported they knew that the baby´s father was also living with another woman compared to 20.6% (85/414) in the older group, although it should be noticed that 48.5% (50/104) of the adolescent girls and 36.1% (149/414) of the older women group preferred not to answer to this question.

Concerning living arrangements, 68% (71/104) of the adolescents were living with their husbands/boyfriend and 28.7% with others: 13.4% (14/104) with the pregnant woman´s mother and 15.3% (16/104) with other relatives; 1.9% (2/104) adolescents mentioned that they were living alone. Three hundred and fifty-three/414 (85%) of the older women were living with their husbands/men, 4.5% (19/414) alone, 4.5% (19/414) with the pregnant woman´s mother, 4.1% (17/414) with other relatives and 2.2% (9/414) were living with a man who was not the baby´s father.

**Fig. 2:**
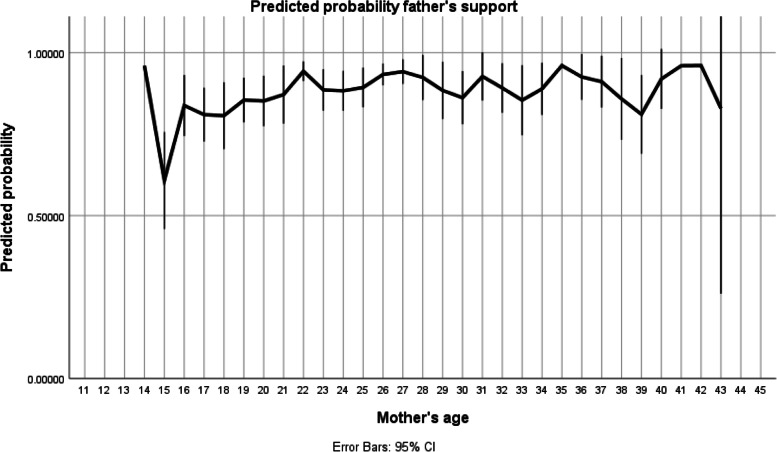
Father´s support according to mother´s age. The line at 0.5 represents equal probability of father’s support during pregnancy

The adolescent´s pregnancy was not supported/followed by the baby´s father (Table 1) in 20% of the cases with a statistically significant difference (OR 0.41, 95% CI 0.2–0.7, *p*=0.002) comparing to the older counterpart. However, after the model was adjusted for marital status, education and antenatal care, the effect of age disappeared totally, as the relevant variable becomes marital status (Fig. 2). In the controlled model, marital status showed a OR 19, with a *p*=0.001.

### Obstetric history and antenatal care

Gravidity, abortions, and number of living children are described in Table 2.

### Previous contraceptive use and future family planning

Previous contraceptive use is described in Tables 2 and 3.

**Table 3:** Future family planning: methods women are willing to use to prevent future pregnancy

	Adolescent group *n*=51 (%)	Older counterpart *n*= 165 (%)
Oral contraceptives	8 (15.6%)	43 (26%)
Injectables	6 (11.7%)	44 (26.7%)
Intra uterine device	3 (5.8)	8 (4.8%)
Sterilization	0	23 (14%)
Condom (male)	0	0
Traditional methods*	34 (66.7%)	32 (19.4%)
OR and 95% CI	0.66 (0.4-1.03)	
*p* value	0.07	

* Rhythm, Calendar, Withdrawal, Lactation amenorrhea

**Fig. 3:**
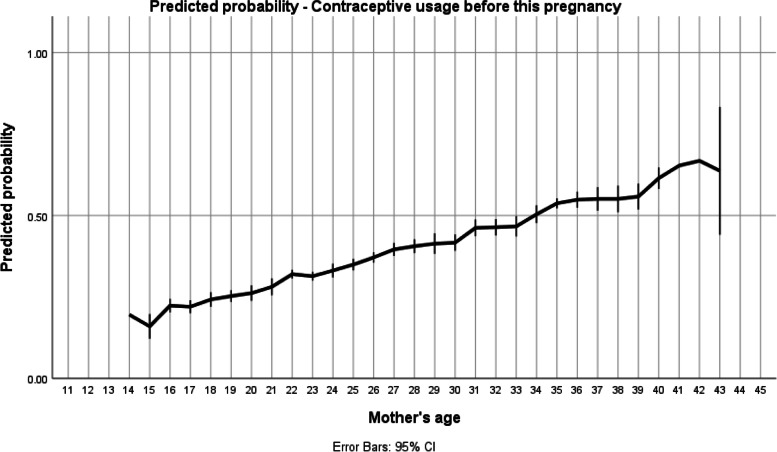
Previous contraceptive use according to women´s age. The model was adjusted for marital status, education, and antenatal care. The line at 0.5 represents equal probability of contraception before birth

When model was adjusted for marital status, education, and antenatal care, the probability of using contraception rises with age, with a turning point at 34 years old (Figure 3), meaning that in Sao Tome and Principe most young women do not assume birth control (Table 2).

Methods women stated that are willing to use to prevent future pregnancy are described in Table 3. Adolescent group most rated (66.7%) preference were for traditional methods (rhythm, calendar, withdrawal, lactation amenorrhea). In this study none of the group referred they would choose the use of a condom by their partner. To note that two older women had an urgent hysterectomy at the time of the current birth and other 7.9% (13/165) performed a sterilization during the caesarean intervention.

### Mothers´ knowledge of identification of newborns’ danger signs

Mothers´ knowledge of identification of newborns’ danger signs was inappropriate in 70.2% (73/104) of the adolescent group and in 13% (54/414) of the older childbearers, being statistically significant (OR 15.7, 95% CI 9–26, *p* ≤0.001).

### Very early, early, and late childbearing adolescents in comparison to older pregnant women

For some variables (Tables 1, 2 and 4) age subgroups were used for the purpose of analysing eventual differences between “very early childbearing” for birth before age ≤15 (n= 8), early childbearing for birth between age >16 and ≤17 (*n*=45) and late childbearing for birth age >18 and ≤19 (*n*=51).

**Table 4:** Standard of living for the different adolescent subgroups in comparison to the older women group

Characteristics	Pregnancies ≤19				Pregnancies >20
	very early childbearing (≤15) *n*= 8 (%)	early childbearing (≥16 and ≤17) *n*=45 (%)	late childbearing (≥18 and ≤19) *n*=51 (%)	Total adolescents ***n***= 104 (%)	***n***=414 (%)
**Electricity**					
No	2 (25%)	12 (27%)	9 (17.6%)	23 (22%)	86 (20.8%)
Yes	6 (75%)	33 (73%)	42 (82.4%)	81 (78%)	317 (76.6%)
missings	-	-	-	-	11 (2.7%)
OR and 95% CI	0.17 (0.08-0.2)	0.69 (0.3-1.4)	1.3 (0.6-2.9)		
*p* value	0.87	0.29	0.43		
**Sanitation**					
open defecation	6 (75%)	31 (69%)	24 (48%)	61 (58.6%)	182 (44%)
latrine	2 (25%)	9 (20%)	24 (48%)	35 (33.6%)	122 (29.5%)
toilet	-	4 (9%)	2 (4%)	6 (5.8%)	94 (22.7%)
missings	-	1 (2%)	1 (2%)	2 (1.9%)	16 (3.9%)
OR and 95% CI	0.32 (0.08-1.22)	0.5 (0.31-0.22)	0.7 (0.4-1.0)		
*p* value	0.09	0.06	0.07		
**Drinking water**					
river	-	4 (9%)	5 (10.4%)	9 (8.6%)	37 (8.9%)
public tap	4 (50%)	8 (18%)	12 (25%)	24 (23%)	109 (26.3%)
protected well	4 (50%)	15 (33%)	12 (25%)	31 (29.9%)	63 (15.2%)
piped water outdoor	-	15 (33%)	16 (33.3%)	31 (29.9%)	152 (36.7%)
piped water indoor	-	3 (7%)	3 (6.3%)	6 (5.7%)	46 (11.1%)
missings	-	-	3 (6.3%)	3 (2.9%)	7 (1.7%)
OR and 95% CI					
*p* value					
**Cooking fuel**					
wood	1 (12.5%)	18 (40%)	16 (31.4%)	35 (33.6%)	127 (30.7%)
charcoal/coal	5 (62.5%)	22 (49%)	31 (60.8%)	58 (55.8%)	235 (56.8%)
gas	2 (25%)	5 (11%)	4 (7.8%)	11 (10.6%)	41 (9.9%)
missing	-	-	-	-	11 (2.7%)
OR and 95% CI	-	0.8 (0.5-1.1)	1.0 (0.6-1.6)		
*p* value	-	0.55	0.98		
**Housing**					
wood	8 (100%)	41 (91%)	46 (90.2%)	95 (91.3%)	328 (79.2%)
mixt	-	3 (7%)	1 (2%)	4 (3.8%)	44 (10.6%)
brick house	-	1 (2%)	4 (7.8%)	5 (4.8%)	31 (7.5%)
missing	-	-	-	-	11 (2.7%)
OR and 95% CI	-	0.5 (0.2-1.2)	0.7 (0.43-1.2)		
*p* value	-	0.13	0.38		

*Abbreviations*: *OR* Odds Ratio, *CI* Confidence interval

Regarding standard of living (Table 4) almost all women in the study were considered as having a deprived standard of living (no electricity and/or open defecation and/or river, and/or cooking wood and coal and/or housing wood or mixt).

A non-statistically significant trend was observed among teenage mothers living in rural areas compared to their older counterpart.

Child marriage was reported in 25% (2/8) of the very early childbearing adolescents and in 69% (31/45) in the early childbearing (birth age >16 and ≤17). When comparing marital status, a statistically significant difference (OR 0.05, 95% CI 0.011-0.276, *p*=0.0001) was found between very early childbearing; (OR 0.35, 95% CI 0.17-0.7, *p*=0.003) early childbearing and (OR 0.464, 95% CI 0.23-0.93, *p*=0.03) late childbearing and older women (Table 1).

With respect to the use of previous contraceptives, these were not used at all in girls ≤15 years, when comparing to 9.8% (5/51) of late childbearing (birth age >18 and ≤19) (Fig. 3 and Table 2).

A total of 14 teenage girls had already experienced a previous abortion: 12.5% (1/8) for very early childbearing; 9% (4/45) in early childbearing subgroup and 18% (9/51) in the late childbearing adolescent. To highlight that 4% of girls between 16 to 17 years old had already one living child. The late childbearing group had already one living child in 25.5% and 5.9% had already two other living children and were expecting the third son before completing her 20^th^ birthday (Table 2).

Regarding differences in the first attendance at ANC facility there was a statistically significant difference between the late childbearing subgroup compared to the older counterpart in attending before the 12th week of pregnancy (OR 2.47, 95% CI 1.24-4.95, *p*=0.01) (Table 2).

## Discussion

Early motherhood is a major public health issue in Sao Tome and Principe that impacts not only girls’ health and future, but also maintains an intergenerational poverty cycle in the country [1, 2]. This study aimed to recognize pregnant adolescent main vulnerabilities to support policy makers in delineating a strategy for preventing the high rates of adolescent pregnancies in the country.

In this study, we found an adolescent pregnancy prevalence of 20.1%, lower compared to the rate of 27% published for the country [26, 27]. This difference can be related to the type of study design of a hospital-based contrarily to a demographic health survey.

A rate of 7.7% of very early adolescent child bearers (14 and 15 years) was identified, a rate two-times higher than the 3.5% published in Africa [3, 21–23] (https://data.unicef.org/topic/child-health/adolescent-health/). Although very early adolescent childbearing is generally rare [41], three of the four countries in the world with more than 10 births per 1,000 girls under 15 years are in sub-Saharan Africa, namely Angola, Mozambique, and Nigeria (https://www.un.org/en/development/desa/population/publications/pdf/popfacts/PopFacts_2019-1.pdf). Our findings suggest that pregnancy under 15 years old is not so rare in Sao Tome and Principe and focus should be given to these biological, social, economic, and emotional particularly more vulnerable girls. In STP, the minimum age at which an individual is considered legally old enough to consent to participation in sexual activity is 14 years old and we didn’t find any girl under this age.

Adolescent girls were particularly more vulnerable concerning to their bond and type of relationship established with the baby´s father due to the reasons next discussed.

First, we found that a high proportion of teenage pregnancies occurred in the first months of the beginning of the union/relationship compared to the older women group (*p*≤0.001), revealing girls’ immaturity and lack of awareness and skills to prevent pregnancy, especially when most adolescent girls stated that the pregnancy was not planned.

Second, previous contraceptive methods were not used at all in girls younger than 15 years old and in most of girls aged between 16 and 19 years. These findings are similar to most studies in Sub-Saharan Africa that report that few sexually active adolescents use contraceptives methods [42–44]. As described in another study, 51% (26/51) of the pregnant adolescent in STP did not use any contraceptive method, mainly because their partners refuse to do so, and the preferred contraceptive method were condoms (41.2%) [31]. Contrarily, to notice that the use of condoms was not an option among the participants of our study.

Third, regarding age-disparate sexual relationships, a big age-gap between sexual partners was found. This age difference, which could be of fifteen years, when referring to very young girls and their partners would be considered sexual abuse in many societies. These relations between women and men translate that women often have less power than their partners in relationships, especially if they are much younger than them [10–13]. One implication of these findings is that pregnant adolescents with older partners are, therefore, a particularly at-risk group and should be prioritized for targeted delivery of sexual reproductive health services. As known, women’s self-efficacy in negotiating condom or other contraceptive use, their capability of sexual communication with their male sex partners and their perceptions of whether their male sex partners were currently having sex with other women is biased and unbalanced, particularly with adolescents and when there is a big age difference between partners [10, 11, 45].

Regarding union or marriage, we found differences between the teenage subgroups, if the 14-15 years old adolescents were mainly single, those aged between 16 and 19 years old were married. These results showed a significant statistical association regarding the status “single” between adolescents and older women group. On the other hand, child marriage, defined as marriage occurring before the age of 18, was founded in a high rate for the early childbearing girls (16-17 years). This reality is well-known in the country with 15.3% of the girls aged 15-19 years to be legally married or in a union; 5.1% before the age of 15 and 32.2% before the age of 18 years [33]. Family Law in the country enables parents to authorize the marriage of children under 18, provided that the woman has already completed 14 years of age and the man has completed 16 years of age [46], what can also promote adolescent pregnancies in the country.

Concerning baby´s father support we initially found a statistically significant difference between groups (*p*=0.002), with the adolescent group in disadvantage. However, taking into consideration the very high prevalence of single parent families in the country [28], the model was adjusted, and the effect of age disappeared totally for father´s support. Other characteristic notable of discussion is that both adolescents and older women acknowledged that the baby´s father was also living with another woman, which is probably associated with a “polygamous” marital informality that is known in the country to be around 22.4% [28]. This can be explained by the fact that in STP, many families live in a kind of non-marital union, which consists of regular or irregular visits by the male spouse to the women with whom he has sexual relations, usually a young woman, with children resulting from this relationship [35, 36].

These culture characteristics can have a particular effect on family planning choices, mainly in contraceptive use and planned pregnancy. For instance, this study demonstrates that unplanned pregnancies are a problem in the country to most women regardless their age and that the probability of using contraception rises with age, with a turning point at 34 years old.

In contrast, some girls reported that they had planned to fall pregnant. The reason why they have planned to be pregnant at such a younger age was not questioned but we suppose that it is due to some culture incentives. In the country there is a saying that “for every union a woman should offer a baby to her men”, what could be related to these planned pregnancies at a younger age [35, 36]. In STP prevails a culture aspect that promotes young girls into believe that, by conceiving a child, they will be bond to their partner having access to a better quality of life and to a social upgrade.

Sexual education and family planning also needs to be reinforced in the country as we can identify some important gaps. For example, among girls who want to prevent a future pregnancy, they prefer using traditional, rather than modern contraceptives that are provided free of charge, implying a poor knowledge of contraception efficacy [28]. In matters of family planning, it should be noticed that 30% of the adolescents enrolled in this study were already in their second or third pregnancy and that 13.4% had experienced a previous abortion, which are similar results to those obtained in other countries and translate a lack of family planning [46]. Family planning services in STP should actively follow-up these high-risk adolescents to prevent further early pregnancies.

On the other hand, missing opportunities during ANC service in STP can be perceived through this study due to lack of knowledge of adolescent girls in the identification of the newborn’s dangers signs in comparison to the older women group, with a statistically significant difference. Therefore, one strategy to overcome this problem is using the ANC attendances to provide, particularly to the teenage pregnant girls, information regarding newborn care and awareness of disease signs and symptoms [47].

School attendance in preventing teenage pregnancies is a key strategy [23, 48, 49]. In our study, educational level was similar among groups and most participants were classified as having elementary school, meaning they didn’t attend secondary school, what is usually done from the age of 12 years. Our data are in accordance with other studies in STP that have shown that only 31.1% of adult women reach a secondary level of education and that female participation in the labour market is 41.3% compared to 75.4% for men [27]. Adolescents who are out of school are denied access to comprehensive sexual education and skills needed to negotiate sexuality and reproductive options and prevent pregnancy [48].

To highlight that most adolescent girls in this study were neither studying nor working at the time they got pregnant. Paradoxically, those who were students, had to dropped out from school due to the government disciplinary act prohibiting pregnant girls in the third month of pregnancy from attending classes or school activities [35, 36]. The same sanction was applied to male students involved in the pregnancies. Recently, in 2020, Sao Tome and Principe took a big step to make its education system more equitable and the government revoked this sanction to pregnant young girls and boys [23, 29, 30].

Enhancing adolescents’ education through school sexual education programmes in the country should be encouraged, as it can boost their knowledge and their skill to make autonomous decisions, to use contraceptives, to plan to start their own family and to be pregnant later on [10, 23, 45].

Delaying adolescent childbearing in Sao Tome and Principe demands focusing on its major underlying factors: debut of first sex, marriage, and effective contraceptive use—along with the social, cultural, and economic background surrounding these behaviours, remarkably girls’ education and gender inequality [50–57].

## Study limitations

This study has some limitations. HAM is a reference hospital located in an urban area. This limits its accessibility to women in rural areas and with low socioeconomic level, thus providing a selection bias. Other significant limitation is that we only identified eight adolescent girls with age ≤15 (very early adolescent childbearing subgroup), although pregnancy in early ages is rare, this small sample could have an impact in the analyses of all the differences between adolescent subgroups.

Other behavioural, social, economic, and family related factors that affect adolescent pregnancy could not be addressed in this study and are a possible future research area, such as: previous family history since adolescent pregnant girls tend to present a positive family history of adolescent pregnancy, mainly in their mothers [8, 12, 18]; girls’ parents’ education and parent to child communication on sexual reproductive health issues [8]. Behaviour and emotional factors towards an early pregnancy as negative feelings, not knowing the facts about sexual risk, expectations, option of abortion, peer pressure, partner reaction, family support, and a declared intent to become pregnant at a younger age should also be further investigate.

As this study addressed a sensitive subject matter, it could have had the potential of making adolescents or older women uncomfortable in answering some questions, therefore, there could have the possibility of some incorrect answers; however, a high level of confidentiality was maintained during the interviews to ensure that pregnant women were at ease in giving their responses.

Confusing and misleading information on basic health issues as safe period, painful menses, puberty maturational problems, emergency contraception and newborn health were mentioned by girls as major problems in other studies and these subjects should be reinforced in health care visits [1].

## Conclusions

Many adolescents in Sao Tome and Principe seem to be experiencing an unplanned pregnancy in the first two months after the beginning of their union/relationship and have a higher probability of not having the baby’s father support, being single, student, having no job and lacking appropriate knowledge of identifying newborns’ danger signs in comparison to older women. Vulnerability issues as having a sexual life with no previous contraceptive use were also observed. Other concerns, raised by this study, are the age gap difference between young pregnant adolescents and their sexual partners, polygamous relationships, previous abortions and having other living children without the intention to future adopt any contraceptive methods.

Lack of family planning was recognized as a major problem for all the participants, regardless of their age. Thus, providing proper family planning in the country should be urgently reinforced, as it is the key for reducing health, social and psychological costs of unplanned pregnancies, mainly in adolescents.

This can only be done by simultaneously implementing school programmes on sexual education targeting boys and girls with raising awareness of birth control responsibility, namely the use of effective contraceptive methods that should be available to them. Promotion of teenagers’ decision-making processes must focus on both genders, and girls’ and boys’ sexual education should be optimized not only in schools but also through media as radio and television programmes or digital content as mobile apps. Another strategy is to implement programs that promote parent-teenage girls and boys communication of reproductive health issues, starting from early ages, in order to build skills to prevent pregnancy in the late teenage years [50].

Since adolescent sexuality is a culturally sensitive issue, policies that acknowledge the needs of each age-group and programs built with adolescent input that are locally appropriate should be encouraged (https://www.un.org/en/development/desa/population/publications/pdf/popfacts/PopFacts_2019-1.pdf).

None of these interventions will be truly successful in Sao Tome and Principe if the government doesn´t improve, meanwhile, girls’ access to adequate living conditions, gender equality and enhance their economic status, ending the cycle of poverty that most are doomed to live and to pass-on to the next generation.

### Contribution of our study to knowledge

No study on this subject has previously been published on the socio-demographic and obstetric history differences between adolescents’ subgroups and comparing them to older women. The proposed study is the first comprehensive in Sao Tome and Principe, integrating a multivariate analysis in explaining who the adolescents at risk for early pregnancy are in Democratic Republic of Sao Tome and Principe.

## Data Availability

The datasets used and/or analysed during the current study are not publicly available in order to ensure participants’ privacy and confidentiality but are available from the corresponding author on reasonable request, with the permission from the Director General of Health, Sao Tome and Principe.

## Abbreviations

**STP**: Democratic Republic of Sao Tome and Principe
HAM: Hospital Dr. Ayres de Menezes
OR: Odds Ratio
CI: Confidence Interval
SDG: Sustainable Development Goal
ANC: Antenatal care
DHS: Demographic and Health Survey
MICS: Multiple Indicator Cluster Survey
WHO: World Health Organization
UNFPA: United Nations Population Fund

## References

[1] Ilika A, Anthony I (2004). Unintended pregnancy among unmarried adolescents and young women in Anambra State, south east Nigeria. African Journal of Reproductive Health..

[2] Nkhoma DE, Lin CP, Katengeza HL, Soko CJ, Estinfort W, Wang YC, Juan SH, Jian WS, Iqbal U (2020). Girls’ empowerment and adolescent pregnancy: A systematic review. International journal of environmental research and public health..

[3] Blum RW, Gates WH. Girlhood, not motherhood: preventing adolescent pregnancy. United Nations Population Fund (UNFPA). 2015.

[4] Marston C, Cleland J (2003). Do unintended pregnancies carried to term lead to adverse outcomes for mother and child? An assessment in five developing countries. Popul Stud (Camb)..

[5] Hajizadeh M, Nghiem S (2020). Does unwanted pregnancy lead to adverse health and healthcare utilization for mother and child? Evidence from low- and middle-income countries. Int J Public Health..

[6] Parsons J, Edmeades J, Kes A, Petroni S, Sexton M, Wodon Q. Economic impacts of child marriage: a review of the literature. Rev Faith Int Aff. 2015;13(3):12–22.

[7] Mekonnen T, Dune T, Perz J (2019). Maternal health service utilisation of adolescent women in sub-Saharan Africa: a systematic scoping review. BMC pregnancy and childbirth..

[8] Kassa GM, Arowojolu AO, Odukogbe AA, Yalew AW (2018). Prevalence and determinants of adolescent pregnancy in Africa: a systematic review and meta-analysis. Reproductive health.

[9] Schipulle U (2015). Adolescent pregnancies in Central Gabon: a description of epidemiology and birth outcomes.

[10] Darroch JE (2016). Adding It Up: Costs and Benefits of Meeting the Contraceptive Needs of Adolescents.

[11] Yakubu, I., Salisu, W.J. Determinants of adolescent pregnancy in sub-Saharan Africa: a systematic review. Reprod Health 15, 15 (2018). https://doi.org/10.1186/s12978-018-0460-4 10.1186/s12978-018-0460-4 PMC5787272 29374479

[12] Hajizadeh M, Nghiem S (2020). Does unwanted pregnancy lead to adverse health and healthcare utilization for mother and child? Evidence from low-and middle-income countries. International Journal of Public Health..

[13] Begley E, Crosby RA, DiCLEMENTE RJ, Wingood GM, Rose E (2003). Older partners and STD prevalence among pregnant African American teens. Sexually transmitted diseases..

[14] United Nations (UN), Adolescent Fertility Since the International Conference on Population and Development (ICPD) in Cairo, New York: UN Population Division, Department of Economic and Social Affairs, 2013. https://www.un.org/en/development/desa/population/publications/pdf/fertility/Report_Adolescent-Fertility-since-ICPD.pdf.

[15] Family Planning 2020, Family Planning 2020: accelerating progress, strategy for 2016–2020, 2015, http://www.familyplanning2020.org/microsite/strategy

[16] Chen, X.K., Wen, S.W., Fleming, N., Demissie, K., Rhoads, G.G. and Walker, M. (2007) Teenage Pregnancy and Adverse Birth Outcomes: A Large Population Based Retrospective Cohort Study. International Journal of Epidemiology, 36, 368-373. http://dx.doi.org/10.1093/ije/dyl284 10.1093/ije/dyl284 17213208

[17] Ganchimeg T, Ota E, Morisaki N, Laopaiboon M, Lumbiganon P, Zhang J (2014). Pregnancy and childbirth outcomes among adolescent mothers: a World Health Organization multi-country study. BJOG..

[18] Hodgkinson S, Beers L, Southammakosane C, Lewin A (2014). Addressing the mental health needs of pregnant and parenting adolescents. Pediatrics..

[19] Harville EW, Madkour AS, Xie Y. Predictors of birth weight and gestational age among adolescents. Am J Epidemiology. 2012;176(suppl_7):S150–63. 10.1093/aje/kws231 PMC3530360 23035139

[20] World Health Organization (WHO). WHO Guidelines for preventing early pregnancy and poor reproductive outcomes in adolescents in developing countries. Geneva: WHO; 2011. ISBN: 978 92 4150221 4. https://www.who.int/publications/i/item/9789241502214.

[21] UNFPA. Girlhood not motherhood. Preventing adolescent pregnancy. New York: UNFPA; 2015.

[22] United Nations Population Fund (UNFPA). The State of World Population. Motherhood in Childhood: Facing the challenge of adolescent pregnancy, vol. 2014. New York: UNFPA; 2013. ISBN 978-0-89714-014-0. https://www.unfpa.org/sites/default/files/pub-pdf/EN-SWOP2013-final.pdf.

[23] Loaiza E, Liang M. Adolescent pregnancy: a review of the evidence. UNFPA. 2013. https://www.unfpa.org/sites/default/files/pub-pdf/ADOLESCENT%20PREGNANCY_UNFPA.pdf.

[24] Azzopardi PS, Hearps SJ, Francis KL, Kennedy EC, Mokdad AH, Kassebaum NJ, Lim S, Irvine CM, Vos T, Brown AD, Dogra S (2019). Progress in adolescent health and wellbeing: tracking 12 headline indicators for 195 countries and territories, 1990–2016. The Lancet..

[25] EWEC. The global strategy for women’s, children’s and adolescents’ health (2016–2030). New York; 2015. http://www.who.int/pmnch/media/events/2015/gs_2016_30.pdf 10.2471/BLT.16.174714 PMC4850547 27147756

[26] Democratic Republic of Sao Tome and Princípe: 2018 Article IV Consultation, Fifth Review Under the Extended Credit Facility Arrangement, Request for Waivers for Nonobservance of Performance Criteria, and Financing Assurances Review International Monetary Fund. African Dept. International Monetary Fund, 2018_ ISBN 1484372255, 9781484372258. https://www.ebooks.com/en-us/book/96331484/democratic-republic-of-s-o-tom-and-princ-pe/international-monetary-fund-african-dept/.

[27] UNDP, UNDP. Human development indices and indicators: 2018 statistical update. 2018. at http://hdr.undp.org/sites/default/files/Country-Profiles/STP.pdf

[28] Instituto Nacional de Estatística, 2016. Inquérito aos Indicadores Múltiplos 2014 de São Tomé e Príncipe, Relatório Final. São Tomé, São Tomé e Príncipe. http://ms.gov.st/wp-content/uploads/2019/01/MICS-Final-Report-STP_Portugu%C3%AAs.pdf.

[29] Franklin C, Reeder M. Pregnant and Parenting Adolescents. Encyclopedia of Social Work. 2016. 10.1093/acrefore/9780199975839.013.1054.

[30] Skatrud JD, Bennett TA, Loda FA (1998). An overview of adolescent pregnancy in rural areas. J Rural Health..

[31] Carvalho F, et al. Teenage pregnancy - A study in São Tomé and Príncipe. International Journal of Adolescent Medicine and Health. 2017;32(1):1–5. 10.1515/ijamh-2017-0088. 10.1515/ijamh-2017-0088 28829756

[32] Sao Tome and Principe WHO statistical profile. WHO Libr. 2015. https://www.who.int/gho/countries/stp.pdf.

[33] World Health Organization (WHO). Global Health Statistics 2014:160–161. https://play.google.com/books/reader?id=_dIXBgAAQBAJ&pg=GBS.PA164&hl=en.

[34] United Nations General Assembly (2015). Resolution adopted by the general assembly on 25 September 2015: transforming our world: the 2030 agenda for sustainable development.

[35] Cohen G (2011). A gravidez precoce: estudo qualitativo sobre conhecimento, atitudes e práticas relacionadas com a sexualidade e gravidez entre os adolescentes e jovens em São Tome e Principe.

[36] Andrade DS, David FC (2015). Conhecimentos, práticas e vivências da sexualidade em jovens são-tomenses. Revista Ibero-Americana de Estudos em Educação.

[37] Diário da República nº. 55 (1977), Lei nº. 2/77 (Lei da Família), 28 de Dezembro de 1977. https://data.unicef.org/wp-content/uploads/2017/12/Lei-n%C2%BA-2-77-Regula-juridicamente-as-instituicoes-de-familia.pdf.

[38] Alkire S, Kanagaratnam U, Suppa N. The global Multidimensional Poverty Index (MPI) 2020. no. 49, Oxford Poverty and Human Development Initiative (OPHI), 2021, pp. 1–37. https://ophi.org.uk/multidimensional-poverty-index/global-mpi-2020/.

[39] The Young Infants Clinical Signs Study Group. Clinical signs that predict severe illness in children under age 2 months: a multicentre study. Lancet. 2008;371:135–42. 10.1016/S0140-6736(08)60106-3 18191685

[40] World Health Organization (2005). Handbook: IMCI Integrated management of childhood illnesses.

[41] Chandra-Mouli V, Ferguson BJ, Plesons M, Paul M, Chalasani S, Amin A, Pallitto C, Sommers M, Avila R, Biaukula KV, Husain S (2019). The political, research, programmatic, and social responses to adolescent sexual and reproductive health and rights in the 25 years since the International Conference on Population and Development. Journal of Adolescent Health..

[42] Jonas K, Crutzen R, Van Den BB, Sewpaul R, Reddy P (2016). Teenage pregnancy rates and associations with other health risk behaviours: a three-wave cross-sectional study among South African school-going adolescents. Reprod Health..

[43] Krugu JK, Mevissen FE, Prinsen A, Ruiter RA (2016). Who’s that girl? A qualitative analysis of adolescent girls’ views on factors associated with teenage pregnancies in Bolgatanga. Ghana. Reprod Health..

[44] Kirby BD (2001). Understanding what works and what doesn’t in reducing adolescent sexual risk-taking. Fam Plan Perspect..

[45] Lloyd C (2006). Schooling and Adolescent Reproductive Behavior in Developing Countries, Millennium Project.

[46] Minko JI, Mimbila-Mayi M, Minto’o S, Mikolo AL, Ngonde L, et al. Study of the Maternal and Neonatal Prognosis during Teenage Childbirth at the University Teaching Hospital of Libreville-Gabon. J Clin Med Ther. 2018;3(2):9. https://www.imedpub.com/articles-pdfs/study-of-the-maternal-and-neonatal-prognosis-during-teenage-childbirth-atthe-university-teaching-hospital-of-librevillegabon.pdf.

[47] Sandberg J, Pettersson KO, Asp G, Kabakyenga J, Agardh A (2014). Inadequate knowledge of neonatal danger signs among recently delivered women in southwestern rural Uganda: a community survey. PLoS One..

[48] Hofferth SL, Reid L, Mott FL (2001). The effects of early childbearing on schooling over time. Family planning perspectives..

[49] Woog V, Kågesten A (2017). The sexual and reproductive health needs of very young adolescents aged 10–14 in developing countries: what does the evidence show.

[50] Ayele BGK, Gebregzabher TG, Hailu TT, Assefa BA (2018). Determinants of teenage pregnancy in Degua Tembien District, Tigray, Northern Ethiopia: A community-based case-control study. PloS one.

[51] Bankole A (2007). Sexual behavior, knowledge and information sources of very young adolescents in four sub-Saharan African countries. African Journal of Reproductive Health.

[52] Darroch JE, Woog V, Bankole A, Ashford LS, Points K. Costs and benefits of meeting the contraceptive needs of adolescents. Guttmacher Institute. 2016. https://www.guttmacher.org/sites/default/files/report_pdf/adding-it-up-adolescents-report.pdf.

[53] Kato-Wallace J (2016). Adolescent Boys and Young Men: Engaging Them as Supporters of Gender Equality and Health and Understanding Their Vulnerabilities, Washington, DC: Promundo; and New York: UNFPA.

[54] Croce-Galis M, Salazar E, Lundgren R (2014). Male Engagement in Family Planning: Reducing Unmet Need for Family Planning by Addressing Gender Norms, Washington.

[55] Denno DM, Hoopes AJ, Chandra-Mouli V (2015). Effective strategies to provide adolescent sexual and reproductive health services and to increase demand and community support. Journal of Adolescent Health.

[56] Gottschalk LB, Ortayli N (2014). Interventions to improve adolescents’ contraceptive behaviors in low- and middle-income countries: a review of the evidence base. Contraception.

[57] Boamah EA, Asante KP, Mahama E, Manu G, Ayipah E, Adeniji E, Owusu-Agyei S. Use of contraceptives among adolescents in Kintampo, Ghana: a cross-sectional study. Open Access J Contracept. 2014;5:7-15. 10.2147/OAJC.S56485.

